# Apnea–hypopnea index decreased significantly after nasal surgery for obstructive sleep apnea: A meta-analysis

**DOI:** 10.1097/MD.0000000000006008

**Published:** 2017-02-03

**Authors:** Jun Wu, Guoqiang Zhao, Yunchuan Li, Hongrui Zang, Tong Wang, Dongbo Wang, Demin Han

**Affiliations:** Department of Otorhinolaryngology Head and Neck Surgery, Beijing Tongren Hospital, Capital Medical University, and Key Laboratory of Otolaryngology Head and Neck Surgery, Ministry of Education, Beijing, P.R. China.

**Keywords:** apnea–hypopnea index, Epworth sleep scale, meta-analysis, nasal surgery, obstructive sleep apnea

## Abstract

**Background:**

Nasal surgeries have been applied to obstructive sleep apnea (OSA) patients with nasal obstruction for decades. However, the efficiency of nasal surgery in improving OSA remains controversial.

The aim of this study was to identify whether isolated nasal surgery can improve apnea–hypopnea index (AHI).

**Methods:**

Computerized searches were performed in MEDLINE, Web of Science, Cochrane Library, and Scopus from January 1, 2000 to April 30, 2016. A total of 18 articles and 587 participants were included. There were 1 randomized controlled trials, 2 nonrandomized trials, 11 prospective studies, and 4 retrospective studies. Data regarding study design (prospective/retrospective clinical trial, randomized, and controlled), population size, participant characteristics (age, gender, and body mass index), surgical intervention, and outcomes (AHI, Epworth sleep scale [ESS]) was collected.

**Results:**

Statistically significant improvement in AHI (subgroup 1: weighted mean difference [WMD] [95%confidence interval (CI)], −4.17 [−7.62, −0.73]; subgroup 2: WMD [95%CI], −4.19 [−7.51, −0.88]; overall: WMD [95%CI], −4.15 [−6.48, −1.82]) and ESS (subgroup 1: WMD [95%CI], −2.14 [−3.08, −1.19]; subgroup 2: WMD [95%CI], −4.70 [−5.95, −3.44]; overall: WMD [95%CI], −4.08 [−5.27, −2.88]) was revealed.

**Conclusion:**

Both AHI and ESS improved significantly after isolated nasal surgery, but the improvement of AHI is slightly significant. Future randomized controlled trials are needed to confirm the long-term benefits of nasal surgery on OSA.

## 1 Introduction

Obstructive sleep apnea (OSA) is characterized by recurrently complete or partial obstruction of the upper airway during sleep, resulting in major cardiovascular and neurocognitive sequelae if not treated.^[1,2]^ The obstruction could occur at multiple levels of the upper airway, such as nasal cavity, pharyngeal cavity, and retroglottal region, among which nasal cavity accounts for 1/2 to 2/3 of the general airway resistance.^[3]^

The relationship between nasal resistance (NR) and sleep disordered breathing has aroused the attention of researchers since 19 century.^[4,5]^ Nasal structure malformation has been related to upper airway collapses in some patients with OSA as one of the principal causes.^[6–8]^ And epidemiological studies indicated that adults with nasal obstruction are more likely to have habitual snoring.^[9,10]^ In addition, acute nasal obstruction in healthy adults such as acute rhinitis can cause sleep disordered breathing.^[11,12]^ Vice versa, the incidence of sleep disordered breathing in patients with nasal septum deviation is far higher than that of normal people.^[13]^ Meanwhile, OSA patients have been proved to suffer from a higher probability of nasal obstruction.^[14,15]^

As one primary treatment for nasal obstruction, nasal surgeries, including septoplasty or/and functional sinus surgery or/and turbinate displacement, as well as nasal cavity ventilation expansion techniques^[16]^ have been applied to OSA patients for decades. However, the efficiency of nasal surgery in improving OSA remains controversial. Two meta-analyses on this topic indicated that nasal surgery can improve Epworth sleepiness scale (ESS) score, which is an indicator of daytime sleepiness. However, apnea–hypopnea index (AHI), which is regarded as a key factor evaluating OSA severity and treatment effect, did not reduce significantly.^[17,18]^

Nevertheless, some newly published articles on this topic showed nasal surgery can decrease AHI in recent 3 years.^[19–22]^ Here, we performed this meta-analysis of studies reporting data of treating OSA with nasal surgery.

## 2 Materials and methods

### 2.1 Information source and search strategy

Computerized and manual searches of 4 databases (MEDLINE, Web of Science, Cochrane Library, and Scopus) were performed from January 1, 2000 to April 30, 2016 to identify all data of relevance. The following keywords and MeSH terms were used: “nasal surgery/sleep disorder,” “nasal surgery/sleep apnea,” “nasal surgery/snoring,” “nose/sleep disorder,” “nose/sleep apnea,” “nose/snoring,” “nasal obstruction/surgery,” “rhinoplasty/sleep disorder,” “septorhinoplasty/sleep disorder” and “turbinectomy/sleep disorder,” “rhinoplasty/sleep apnea,” “septorhinoplasty/sleep apnea” and “turbinectomy/sleep apnea,” “rhinoplasty/snoring,” “septorhinoplasty/snoring,” and “turbinectomy/snoring.” The cited references in the relevant articles were also reviewed to identify additional published work. Two reviewers conducted the searches independently, and duplicates were excluded. A 3rd reviewer would resolve disagreements by discussion.

### 2.2 Eligibility criteria and study selection

Articles were screened by titles and abstracts then reviewed if full texts were eligible. Inclusion criteria for the studies consisted of: patients with OSA; isolated nasal surgery applied, such as septorhinoplasty, rhinoplasty, turbinectomy, or sinus surgery; both post- and preoperative quantitative outcomes data evaluating AHI/ESS; and articles published only in English. Studies were excluded for the following criteria: age < 18 years old; case reports, letters to the editor, and review articles; and additional level surgery described (tonsillectomy, uvulopalatopharyngoplasty, maxillomandibular advancement, etc.).

### 2.3 Data extraction

Data regarding study design (prospective/retrospective clinical trial, randomized, and controlled), population size, participant characteristics (age, gender, and body mass index [BMI]), surgical intervention, and outcomes (AHI, ESS) were collected. Two authors independently checked the data to ensure accuracy. Disagreements were resolved by discussion with a 3rd author.

### 2.4 Data analysis

The statistical analysis was performed with IBM SPSS Statistics software version 18.0 (Chicago, IL) and the Cochrane Collaboration's Review Manager (REVMAN) Software version 5.2. We calculated the means, standard deviations (SDs), and 95% confidence intervals (CIs). The weighted mean differences (WMDs) of AHI and ESS were obtained according to the differences of post- and preoperative values from the original articles. A correlation coefficient between intervention effect and baseline AHI/ESS of a study was calculated as described in Cochrane Handbook for Systematic Reviews of Interventions,^[23]^ in which SD of change was provided. Corr_E_ = (SD^2^__E baseline_ + SD^2^_E final_ − SD^2^__E change_)/(2 × SD_E baseline_ × SD_E final_). And subsequently, the average correlation coefficient between intervention effect and baseline AHI or ESS was applied to impute the SD of change for AHI or ESS in studies of which the SDs of change were not provided. For the current meta-analysis, the average correlation coefficient between intervention effect and baseline AHI was 0.667 among the 6 studies with SD of change data, while the average correlation coefficient or ESS was 0.638 in the 3 studies with related information.

We divided the studies into 2 subgroups (subgroup 1, subgroup 2) according to SD of change while conducting the meta-analysis. Forest plots were graphically inspected, and Cochran Q test (*P* < 0.1, a significant difference between studies) and *I*^2^ statistic (low: 25%, moderate: 50%, and high: 75%) were applied for determining heterogeneity. We used fixed effects model for pooling effects if no or low heterogeneity of treatment effects was found and a random effects model if moderate or high heterogeneity was found.

### 2.5 Ethical approval

This is a meta-analysis about literatures; therefore, ethical approval was not necessary.

## 3 Results

### 3.1 Study characteristics

**Figure 1:**
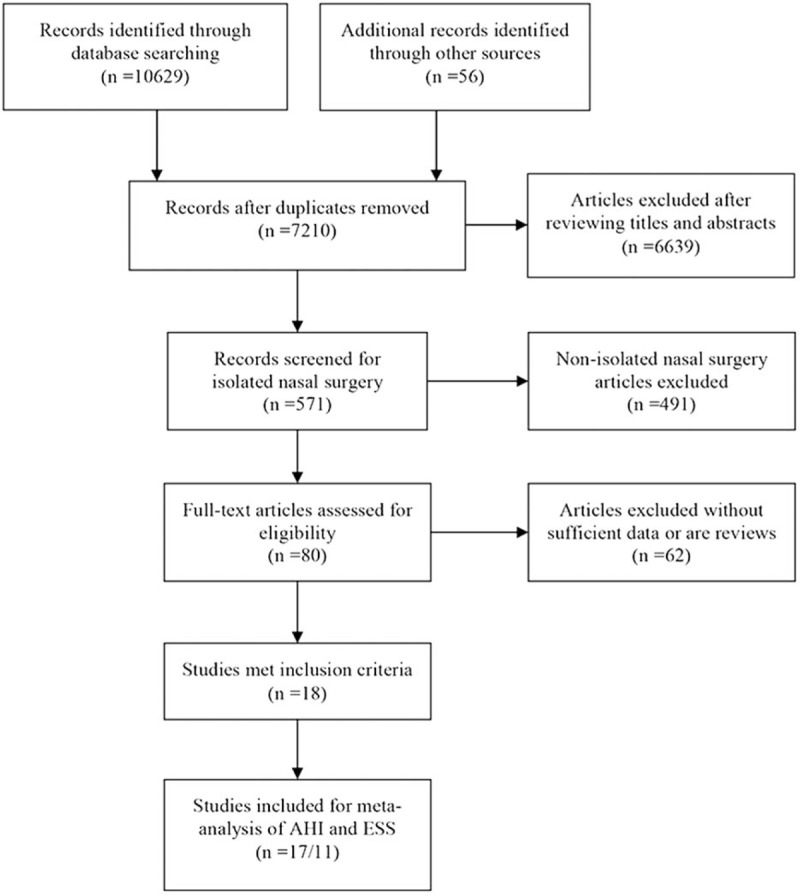
Preferred Reporting Items for Systematic Reviews and Meta-Analyses (PRISMA) flow chart.

Eighteen studies dealing with nasal surgery for OSA met our inclusion criteria, and they included 587 participants.^[19–22,24–37]^ The article selection flow chart is shown in Fig. 1. Overall, 1 randomized controlled trial, 2 nonrandomized controlled trials, 11 prospective studies, and 4 retrospective studies were included. The characteristics of the studies are shown in Table 1. The types of nasal surgery performed in the studies are as the following: septorhinoplasty/septoplasty/submucosal septal resection/septal surgery in 15 studies,^[19,21,22,24,26–28,30–37]^ inferior turbinectomy/partial inferior turbinectomy/submucosal turbinectomy/concha cauterization/turbinate surgery/radiofrequency ablation of inferior turbinate/turbinoplasty in 14 studies,^[19,21,22,24,26–28,30–36]^ and endoscopic sinus surgery in 7 studies.^[19–21,26,27,29,32]^ The treatment protocols are shown in Table 1.

**Table 1:**
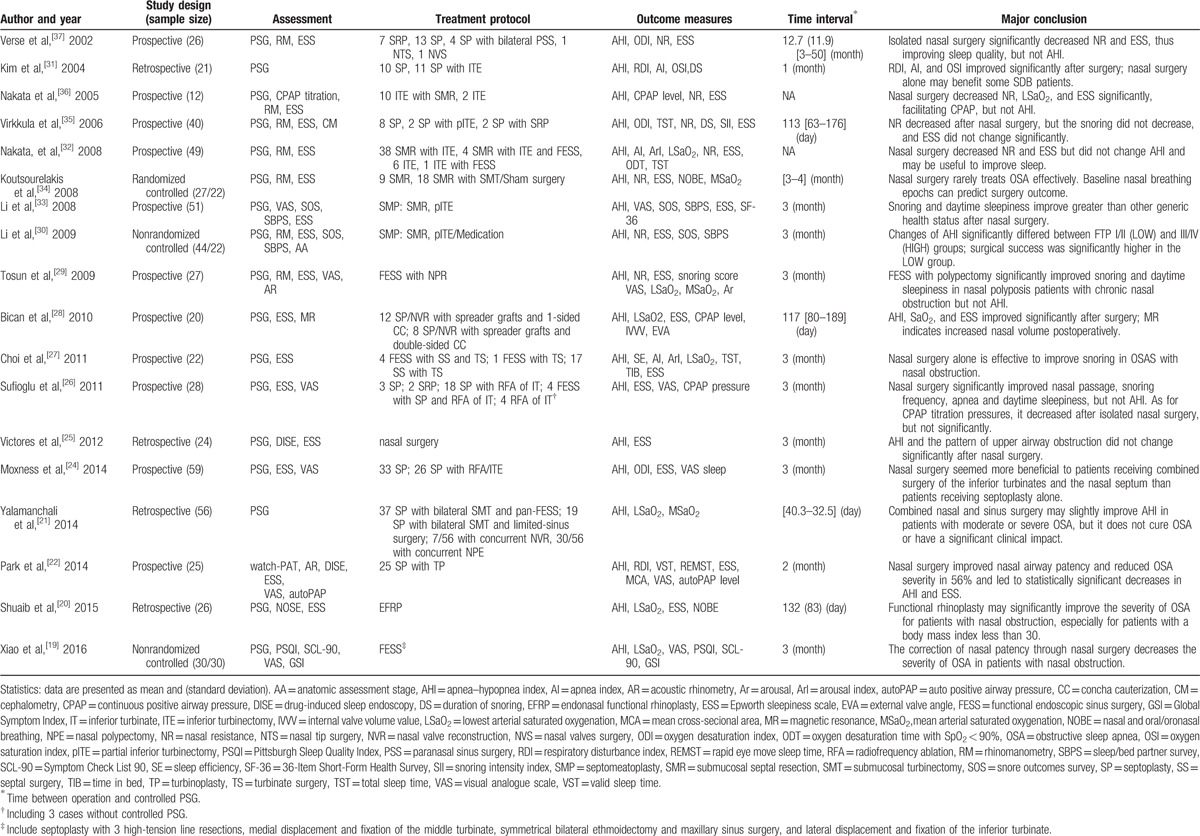
Study characteristics.

Regarding the sleep apnea severity assessment, polysomnography (PSG) was performed in 17 studies,^[19–21,24–37]^ watch-PAT in 1 study.^[22]^ The most commonly used subjective assessment is ESS, which is performed in 16 studies.^[20–22,24–30,32–37]^

### 3.2 Patients characteristics

The age of participants ranged from 20 to 70 years, with an average age of 44 years. The overall proportion of male patients was 90.5%, ranged 63.0% to 100%. Reported baseline BMI, but only 8 studies (44.4%) reported postoperative BMI. The detailed data are shown in Table 2.

**Table 2:**
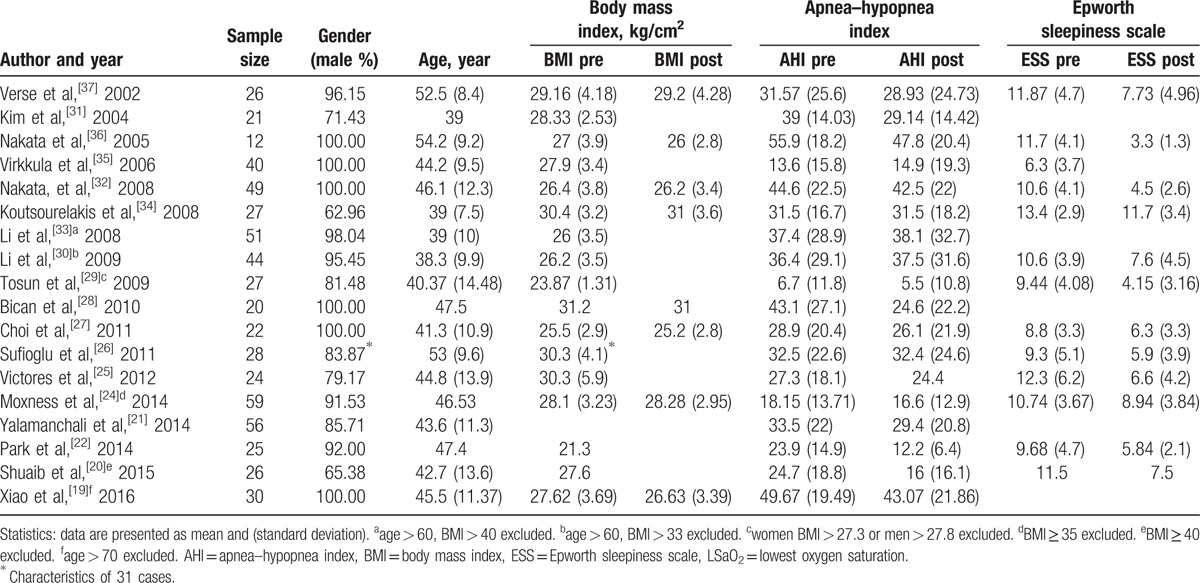
Patients characteristics.

### 3.3 Treatment outcomes

**Figure 2:**
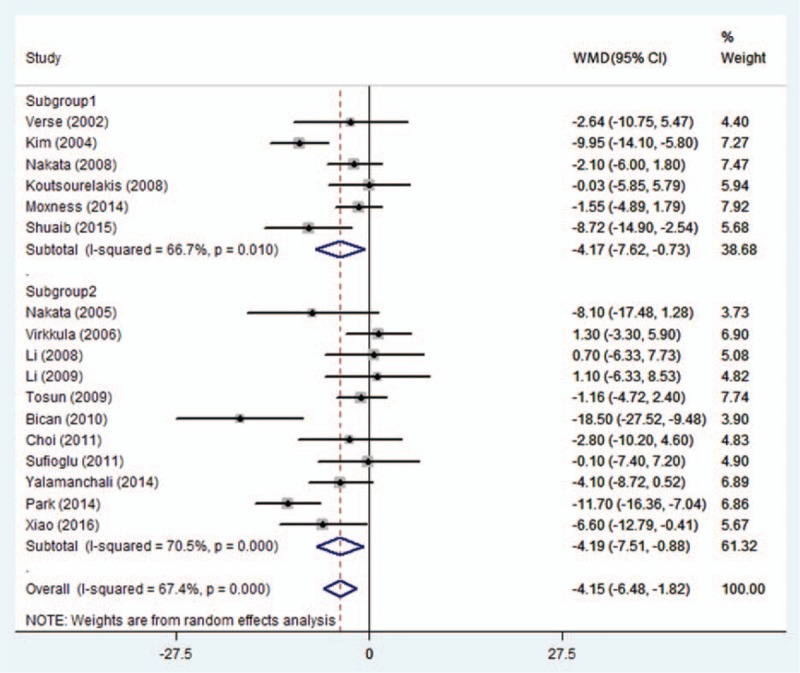
Subgroup 1 includes studies providing AHI SD of outcome parameter; subgroup 2 includes studies not providing AHI SD of outcome parameter. AHI = apnea–hypopnea index, SD = standard deviation.

The study by Victores and Takashima^[25]^ was not included in the analysis of AHI change because it did not provide the SD of outcome parameter. Thus, 17 studies were analyzed. Heterogeneity test results of AHI (subgroup 1: *I*^2^ = 66.7%, *P* = 0.010; subgroup 2: *I*^2^ = 70.5%, *P* = 0.000; overall: *I*^2^ = 67.4%, *P* = 0.000) scores indicate heterogeneity in the studies. Random effects model therefore was used to conduct the meta-analysis. According to the meta-analysis results, statistically significant improvement in AHI (subgroup 1: WMD [95%CI], −4.17 [−7.62, −0.73]; subgroup 2: WMD [95%CI], −4.19 [−7.51, −0.88]; overall: WMD [95%CI], −4.15 [−6.48, −1.82]), Fig. 2.

Five studies were not included in the ESS score analysis since they did not obtain detailed post- or preoperative mean ± SD of ESS scores.^[20,21,28,33,35]^ Thus, ESS scores were only analyzed in the other 11 studies.^[22,24–27,29,30,32,34,36,37]^

**Figure 3:**
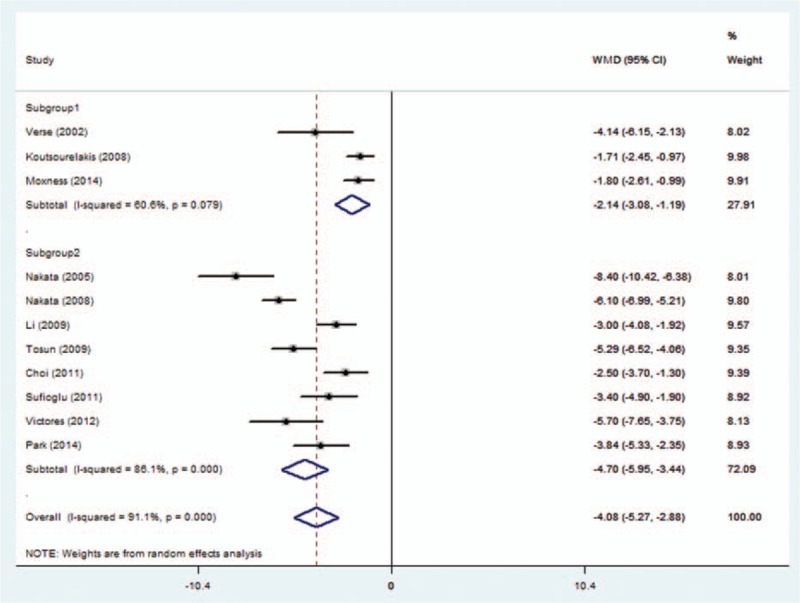
Subgroup 1 includes studies providing ESS SD of outcome parameter; subgroup 2 includes studies not providing ESS SD of outcome parameter. ESS = Epworth sleepiness scale, SD = standard deviation.

Heterogeneity test of ESS showed heterogeneity in the studies (subgroup 1: *I*^2^ = 60.6%, *P* = 0.079; subgroup 2: *I*^2^ = 86.1%, *P* = 0.000; overall: *I*^2^ = 91.1%, *P* = 0.000). ESS decreased significantly, indicating improved day time sleepiness in these patients (subgroup 1: WMD [95%CI], −2.14 [−3.08, −1.19]; subgroup 2: WMD [95%CI], −4.70 [−5.95, −3.44]; overall: WMD [95%CI], −4.08 [−5.27, −2.88]), Fig. 3.

## 4 Discussion

Although different interventions aimed at multiple airway levels have been applied for airway collapse, isolated nasal surgery is rarely considered for the specific management of OSA. However, the reported effects were inconsistent. The present study showed that AHI could be significantly improved postoperatively by isolated nasal surgery. Our analysis used a subgroup analysis, the index before and after the operation according to the reliability of the group, and once again combined to get the same positive results, which provided a more validated result.

The present study included studies evaluating the efficiency of isolated nasal surgery on OSA from 2002 to 2016 and draws a conclusion that both ESS and AHI improved significantly. Our results differed from previous meta-analysis of the efficiency of nasal surgery for OSA. In 2011, a systematic review by Li et al included studies published from 1999 to 2009, and concluded that nasal surgery could effectively improve daytime sleepiness (evaluated by ESS) while not the AHI.^[18]^ A more recent systematic review and meta-analysis in 2015 by Ishii et al,^[17]^ in defect of endoscopic sinus surgery, revealed a similar result that ESS improved significantly, but AHI did not. However, Park et al and some other researchers^[19,20,22]^ reported AHI improvement in selected patients recently.

We attributed the varying conclusions with previous studies to: we included more recent studies accessing sleep apnea severity with a similar criterion; we adopted a more reliable analyzing measurement, which subgrouped and analyzed the collected data. As a measure of surgical intervention, nasal surgery first opens middle nasal meatus and sinuses, therefore helping in draining of nasal and sinus cavity secretion and maintaining their normal physiological function; second decreased upper airway resistance reduces episodes of mouth breathing, negative pressure of the nasopharynx and improves the collapsibility of the oropharyngeal cavity. It is reported that nasal surgeries improved the compliances of continues positive pressure therapy.^[38,39]^ In addition, improvement of psychological symptoms, such as depression as well as daytime sleepiness, were reported even without substantial AHI change.^[30,32–34,36,37]^ Because of the subjective clinical effect on patients, correcting nasal obstruction is still considered an important measure of treating OSA. But the long-term improvement of both objective and subjective indexes needs to be confirmed by a more long-term observational experiment.

The present study compared the difference between post- and preoperative values to evaluate the treatment effect. We did not perform the sensitivity analysis by removing each study to investigate its effect on the summarized effect and heterogeneity. This is because while collecting and organizing data we performed imputation of SD of change in some studies, and we subgrouped and analyzed the included studies basing on whether they provided original individual data or SD of change or we estimated SD of change. Then the subgroups were combined and analyzed. In the meta-analysis of AHI, the result of subgroup 1 is −4.17(−7.62, −0.73), subgroup 2: −4.19(−7.51, −0.88), overall: −4.15(−6.48, −1.82), which indicated consistence of the 2 subgroups with the correlation coefficient 0.667. In the meta-analysis of ESS, similar results were obtained by aforementioned 2 meta-analyses.

In previous studies, researchers regarded increased NR as a contributor in inducing and aggravating OSA, and made conclusions that nasal surgery could decrease NR significantly and improve other sleep indices such as oxygen saturation nadir, arousal index, sleep efficiency, and sleep architecture.^[40]^ However, AHI did not show significant change, with some studies even reporting aggravated AHI postoperatively.^[38]^ A possible explanation is that AHI along, as an indicator of how respiratory events occur, is not sufficient to describe the immigration of sleep stage, or decreases in ventilation (ie, length of the events or the fraction of events that are hypopneas).^[41,42]^ Some other studies reported opposite conclusion that nasal surgery can significantly improve AHI,^[20,22]^ which may result from the different phenotypes of the patients. Multiple risk factors are reported to contribute to sleep apnea, such as abnormal anatomy of upper airway, unstable breathing control, compromised upper airway muscle activation, and low arousal threshold. These individual key factors could affect nasal surgery efficacy. Moreover, the efficiency of unilevel upper airway surgery may eliminate overtime,^[35]^ which might be a reason for the discrepancy of the surgical outcomes.

However, there are still some limitations of this study:

(1) Evidence level of the included literature is different. Only 1 article is level 1, 2 articles are level 2, and other articles are levels 3–4. Study design is relatively rough, not grouping patients according to the severity of disease or preoperative obstructive plane location.
(2) The sample size is small, with the largest size for 59 cases.^[24]^
(3) Surgical methods are not unified, and the selection of symptoms suitable for the surgery is not the same.
(4) Criterion evaluating sleep breathing inconsistent.
(5) There is a certain heterogeneity.

In addition, most of the studies were followed up for a short time, which may have a particular effect on the outcome.

## 5 Conclusion

Both AHI and ESS improved significantly after isolated nasal surgery, but the improvement of AHI is slightly significant. The results of this study provide some evidence supporting for isolated nasal surgery in OSA patients especially those with nasal obstruction. However, in the future, there still should be more randomized clinical controlled trials with multicenter cooperation and a long-term follow-up to evaluate the efficacy of nasal surgery on OSA.
